# Improving diagnostic performances of peanut allergy using novel molecular components

**DOI:** 10.1186/2045-7022-4-S2-P43

**Published:** 2014-03-17

**Authors:** Christelle Richard, Nicolas Couturier, Olivier Roitel, Gunther Knierim, Benoit Thouvenot, Catherine Astier, Françoise Codreanu, Gisèle Kanny, Denise-Anne Moneret-Vautrin, Bernard Bihain, Sandrine Jacquenet

**Affiliations:** 1 GENCLIS SAS, Allergy Department, Vandoeuvre-Les-Nancy, France; 2 Hycor, Allergy Department, Kassel, Germany; 3 Centre Hospitalier de Luxembourg, Food Allergy Department, Luxembourg, Luxembourg; 4 Nancy Hospital, Food Allergy Department, Nancy, France; 5 Epinal Hospital, Food Allergy Department, Epinal, France

## 

Accurate in-vitro component-based diagnosis will allow clinicians to focus their efforts toward complex issues such as disease severity and reactive dose evaluation as well as establishing effective eviction measures. However to achieve this, clinical utility of component-resolved peanut allergy testing must be rigorously evaluated. In this pilot study, we compared performances of 2 component-resolved peanut allergy assays from Hycor**®** [H] and Thermo Scientific**®** [T] that are based on recombinant allergens (rAra h 1 [H/T], rAra h 2 [H/T], rAra h 3 [H/T], rAra h 6 [H], rAra h 7 [H], rAra h 8 [H/T] and rAra h 9 [H/T]). IgE reactivity to each allergen was measured in 34 clinically characterized patients: 26 peanut-allergic and 8 atopic subjects from Eastern and Northern France. Total peanut extract assay [T] yielded low performances even with a 0.35 kU/L cut-off (sensitivity: 92%, specificity: 25%). Quantifications of IgE to rAra h 1, rAra h 2 and rAra h 3 show a strong correlation between the two manufacturers (rAra h 1: r2 = 0.85, p < 10-3 ; rAra h 2: r2 = 0.96, p < 10-3; rAra h 3: r2 = 0.90, p < 10-3) and achieve the same level of performances independently of the allergen source. Among these three assays, rAra h 2 [H/T] yielded a sensitivity of 85% and a specificity of 100% with a 0.35 kU/L cut-off. The rAra h 6 assay is available from Hycor**®** only and yielded a sensitivity of 92% and a specificity of 100% using the same cut-off, i.e. 24/26 peanut allergic patients are positive for rAra h 6. The IgE from the 2 undiagnosed patients do not react with any of the other recombinant from any sources. Neither Hycor**®** nor Thermo Scientific**®** rAra h 8 assays are clinically useful for peanut allergy diagnosis in spite of their specificity differences at 50% and 25% respectively. Thermo Scientific**®** rAra h 9 assay was not clinically useful with no positive sample detected. Hycor**®** rAra h 9 assay detected 2 of the 26 peanut allergic patients without reactivity in the atopic patients (specificity: 12%, sensitivity: 100%). This is consistent with the low prevalence of Ara h 9 sensitisation in Northern Europe. This study suggests the possibility of improving the clinical utility of molecular component assays by substituting rAra h 6 to rAra h 2. It also introduces rAra h 9 as a novel component that might be a significant clinical value in countries outside Northern Europe. This will be evaluated in multicenter studies.

